# Statistical Analysis of Pseudoexfoliation Syndrome Prevalence, Glaucoma and Coronary Artery Disease of the Patients with Cataract

**Published:** 2011-03

**Authors:** Yong Xu, Nicolaos Cosmas, Sotirios Gartaganis

**Affiliations:** 1 *Department of Mathematics and statistical, University of South Florida (USF), USA;*; 2 *Technological Educational Institute of Mesologgi, TEI, Greece;*; 3 *University of Patras, Greece*

**Keywords:** statistical modeling, logistic regression, pseudoexfoliation syndrome prevalence, glaucoma, coronary artery disease and cataract

## Abstract

Cataract is a common disease of the eye and cataract development is usually a very gradual process of normal aging. Many people with cataract disease can also have pseudoexfoliation (PEX) syndrome, glaucoma and coronary artery disease (CHD). We use the data from the Department of Ophthalmology, Medical School of the University of Patras, Greece. These data contains 2140 Greek patients from the southwestern Greece with cataract disease. In order to investigate the association between any two variables statistical analysis were studied. Considering the binary behavior of the response we decide to use non parametric analysis in this study to deal with the testing the populations mean with different distributions.

## INTRODUCTION

Andrikopoulos and Gartaganis in 2008 investigate (2), the prevalence of Glaucoma and Coronary Artery Disease (CAD) in patients with cataract and Pseudoexfoliation (PEX) syndrome. For that they use the sample data of 2140 consecutive patients with cataract admitted at the University Hospital of Patras, Greece, for cataract surgery. Only patients with senile cataract were included in this study. All patients underwent a complete ophthalmological examination that included slit-lamp evaluation with dilated pupil for PEX material in the anterior segment, intraocular pressure (IOP) measurements and optic disc cup examination. They also underwent an evaluation for CAD by a cardiologist. CAD was considered present if a patient had a history of myocardial infarction, or ischemia, or abnormal coronary angiography. The patients were classified into two groups: the PEX and the non-PEX group.

The authors had the following results in (2). One thousand and eighty-eight (50.8%) patients were men and 1052 (49.2%) were women. The overall prevalence of PEX syndrome was found to be 27.9% and it was found to increase with progressing age. Bilateral PEX was more frequent than unilateral PEX, with the percentage of bilateral PEX raising with progressing age. A total of 132 patients (22.1%) in the PEX group exhibited glaucoma, while in the non-PEX group only 2.6% suffered glaucoma. PEX was also found to be positively associated with the risk for CAD among subjects 50 years or older. No association between CAD and glaucoma was found. They conclude that PEX syndrome constitutes a major glaucoma risk factor and a (probable) CAD risk factor. PEX is also positively associated with CAD. Patients with PEX should be informed and examined frequently as the risk is present throughout.

In this paper we consider the same sample data and we try a further statistical analysis. The Goal is to analysis the relationship between PEX, Glaucoma and Coronary Heart Disease (CHD) with all other variables. CHD is another name for CAD. We will start with identify the probabilistic distribution for each disease then we will compare the mean response of the age of patients in male and female group. In the end, we will construct some statistical model so that we can perform certain risk prediction or forecasting for the patients with cataract disease. Some other important and recent references for the readers who will have an interest in eye disease are in (1, 3-7, 11-15).

## PARAMETRIC ANALYSIS

### Descriptive statistic analysis

**Figure 1:**
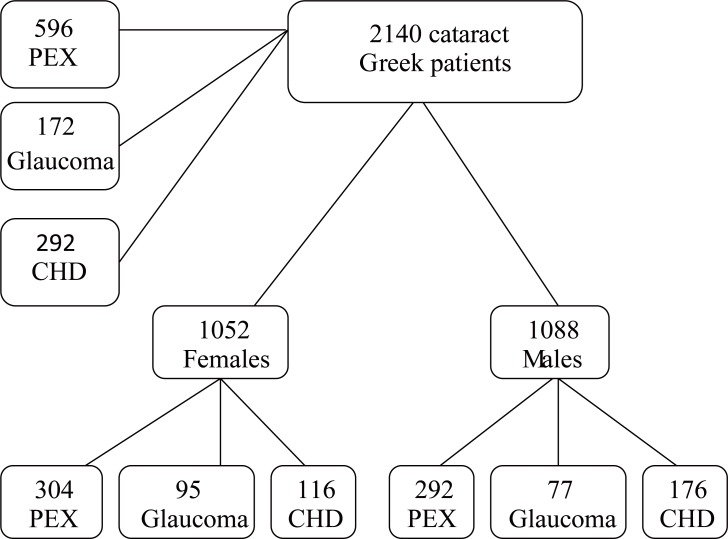
Tree diagram for the Greek patients with cataract.

From Figure 1, below, we start presenting the sample data. As we can see we have 2140 Greek patients with cataract and among them we have 596 patients have PEX, 172 patients have Glaucoma and 292 patients have CHD disease. In the sample data the percentage of the cataract patients to have PEX is 27.85%, to have Glaucoma is 8% and to have CHD is 13.64%. From the descriptive statistics we can figure out that people who have cataract has more chance to have PEX than CHD and the least chance to have Glaucoma.

There are 1052 female patients with cataract and 1088 male patients with cataract. Within female group there are 304 patients have PEX, 95 patients have Glaucoma and 116 patients have CHD disease. The percentage of the female cataract patients to have PEX is 28.9%, to have Glaucoma is 26.84% and to have CHD is 11%. From these descriptive statistics we can find out that female with cataract have more chance to have PEX than Glaucoma and least chance to have CHD. This result is not same as we observed from previous result. That means woman with cataract have more chance to have Glaucoma than man.

Within male group there are 292 patients have PEX, 77 patients have Glaucoma and 176 patients have CHD disease. The percentage of the female cataract patients to have PEX is 26.84%, to have Glaucoma is 7% and to have CHD is 16.18%. From these descriptive statistics we can conclude that male with cataract have more chance to have PEX than CHD and least chance to have Glaucoma. This result is about same with the whole group therefore we can tell that male with cataract have more chance to have CHD than female with cataract and less chance to have PEX than female with cataract.

### Probability structure

We are interested to know the probability structure of these three diseases, namely, PEX, Glaucoma and CHD. We will perform goodness of fit test for all these three diseases. We will mainly focus on Kolmogorov-Smirnov (KS) test’s result and we will consider Anderson-Darling test and Chi-Squared goodness of fit test’s result.

Cauchy distribution is named after Augustin Cauchy. Cauchy distribution is widely used in mathematics and physics among other science subjects. Cauchy distribution’s probability density function (PDF) is as follows:

(a) fx=πσ 1+x−μσ2−1 for   x∈R

where σ is scale parameter and μ is location parameter.

The cumulative probability distribution (CDF) is given by:

(b) $$F\left(x\right)=\frac{1}{\pi }\arctan\left(\frac{x-\mu }{\sigma }\right)+0.5$$

**Figure 2:**
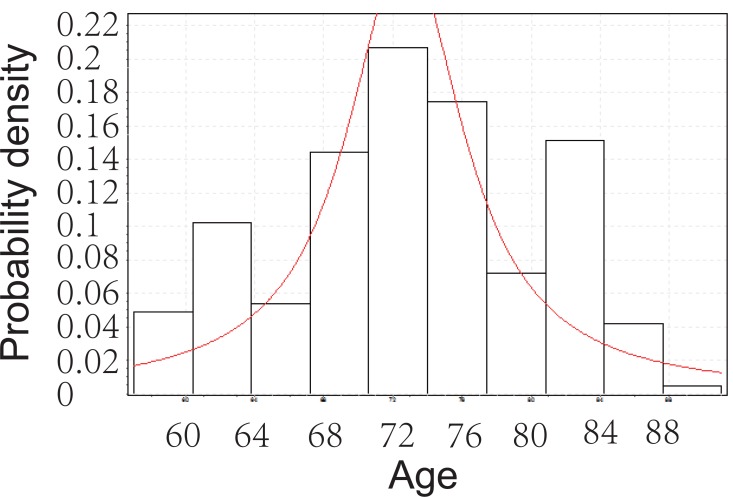
Goodness fit test for PEX patients with cataract.

From Figure 2, below, we can tell that the age of PEX patients with cataract follows Cauchy distribution. The Cauchy pdf is give by:

(c) fx=4.1043π 1+x−72.7064.10432−1

The Cauchy CDF is given by equation (d), below:

(d) $$F\left(x\right)=\frac{1}{\pi }\arctan\left(\frac{x-72.706}{4.1043}\right)+0.5$$

**Figure 3:**
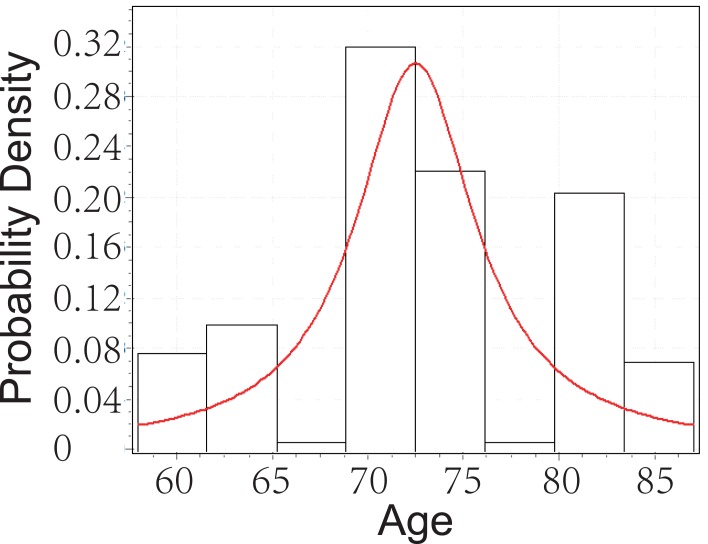
Goodness fit test for Glaucoma patients with cataract.

From Figure 3, below, we can tell that the age of Glaucoma patients with cataract follows Cauchy distribution with parameters *σ*=3.7658 and *μ*=72.483. Although the *p*-value from KS test is 0.00366, which is significant enough, we can still observe the distribution fit is not that good. This will be explained further in later chapter’s analysis.

**Figure 4:**
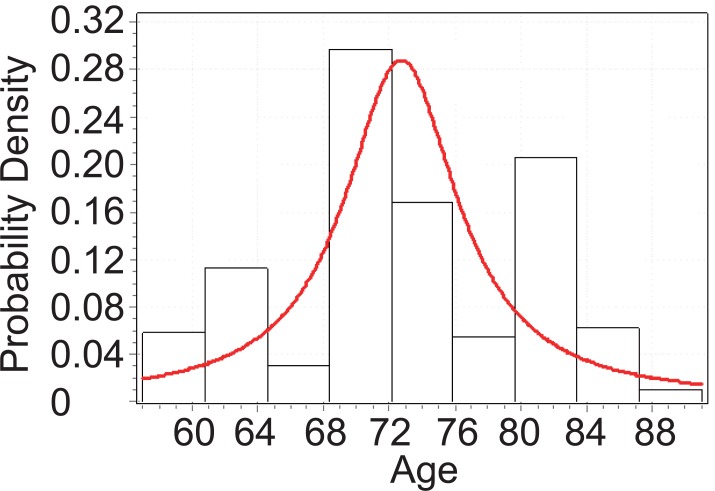
Goodness fit test for CHD patients with cataract.

From Figure 4, below, we can tell that the age of CHD patients with cataract follows Cauchy distribution with parameters *σ*=4.169 and *μ*=72.716.

We can tell that all these three diseases follow the same distribution Cauchy with different parameters. Then we will look through the distribution for both female and male.

**Figure 5:**
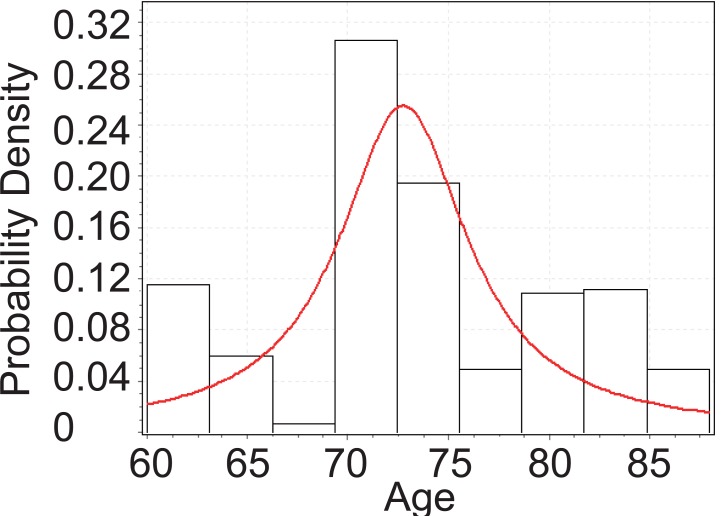
Goodness fit test for female PEX patients with cataract.

From Figure 5, below, we can tell that the age of female PEX patients with cataract follows Cauchy distribution with parameters *σ*=3.8787 and *μ*=72.755.

**Figure 6:**
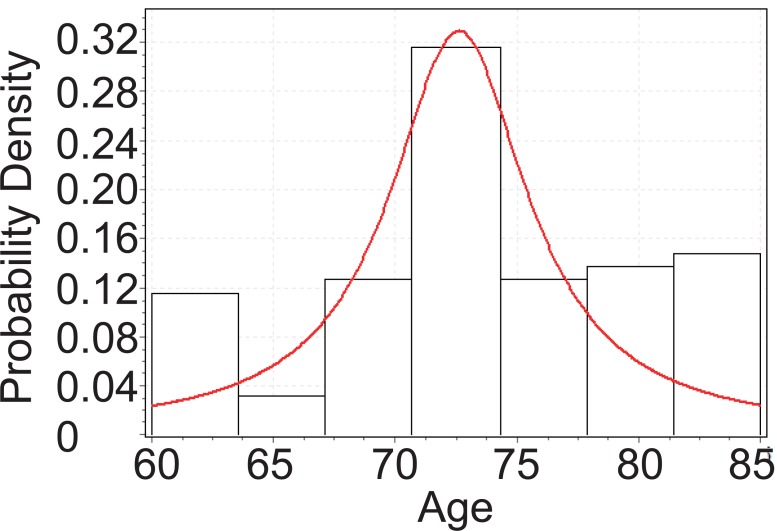
Goodness fit test for female Glaucoma patients with cataract.

From Figure 6, below, we can tell that the age of female Glaucoma patients with cataract follows Cauchy distribution with parameters *σ*=3.4543 and *μ*=72.605.

**Figure 7:**
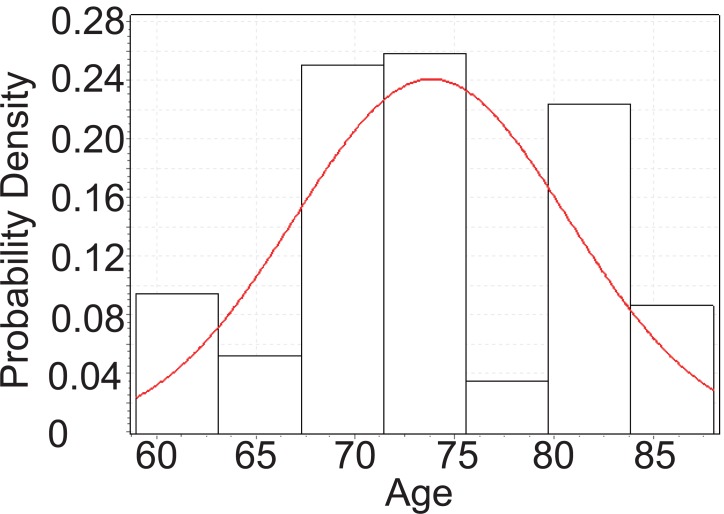
Goodness fit test for female Glaucoma patients with cataract.

From Figure 7, below, we can tell that the age of female CHD patients with cataract follows Normal distribution with parameters *σ*=6.8668 and *μ*=73.828.

We can see for female patients with cataract both PEX and Glaucoma follows Cauchy distribution which are same as the whole patients group’s result while CHD follows normal distribution which are different from the whole patients group’s result.

**Figure 8:**
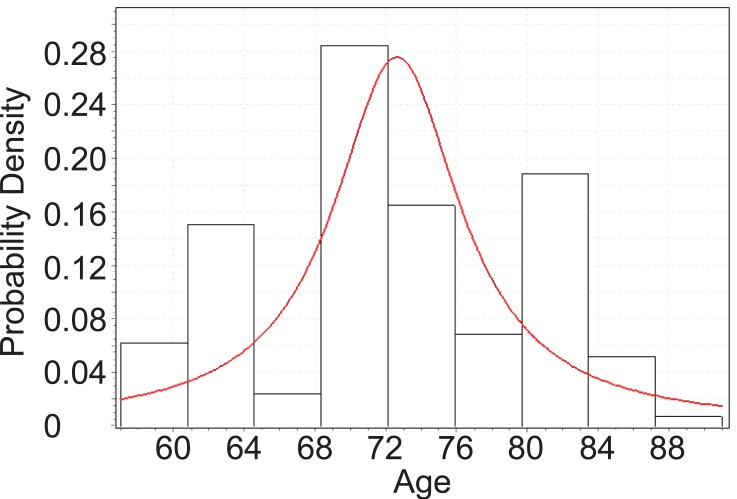
Goodness fit test for male PEX patients with cataract.

Next, we will do the similar goodness of fit test for male patients’ group. From Figure 8, below, we can tell that the age of male PEX patients with cataract follows Cauchy distribution with parameters *σ*=4.3617 and *μ*=72.631.

**Figure 9:**
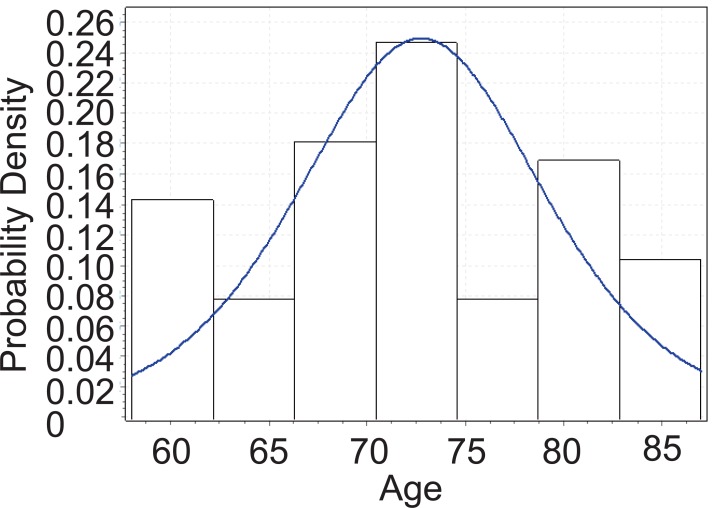
Goodness fit test for male Glaucoma patients with cataract.

Figure 9, below, we can tell that the age of male Glaucoma patients with cataract follows logistic distribution with parameters *σ*=4.1484 and *μ*=72.74.

**Figure 10:**
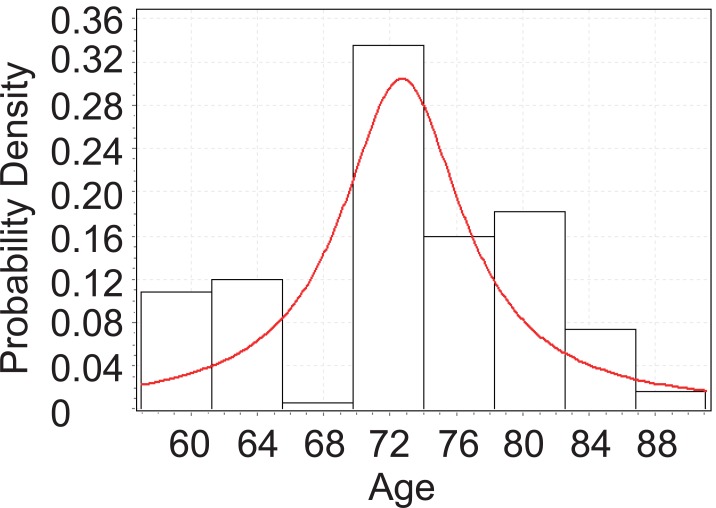
Goodness fit test for male CHD patients with cataract.

From Figure 10, below, we can tell that the age of male CHD patients with cataract follows Cauchy distribution with parameters *σ*=4.4396 and *μ*=72.686.

We can say for male patients with cataract both PEX and CHD follows Cauchy distribution which are same as the whole patients group’s result while Glaucoma follows logistic distribution which are different from the whole patients group’s result. Both male and female patients with cataract’s PEX follow Cauchy distribution but it is not true for CHD and Glaucoma.

## MEAN ANALYSIS

### Two sample t-test

We would like to know if the mean age responses in both male and female patients groups are equal. The first candidate test is independent two sample t-test. Here we will assume both sample data follows normal distribution although we know they are not from the previous analysis. We will perform classical t-test then we will utilize nonparametric analysis. In order to perform independent two sample t-test, we have to meet two conditions. 1) The two samples have to be independent; 2) The two sample variances have to be the same. Condition 1 is easy to satisfy since we get the result from male and female and they are independently chosen. For condition 2, since the two samples have different sample size, we will perform levene test for equality of variances. For PEX patients with cataract the levene test’s *p*-value is 0.1651 therefore we have 95% confidence to not reject null hypothesis this concludes that the two sample’s variance are same. Since both assumptions are satisfied, we will perform two sample t-test.

From Table 1, we can conclude that the two sample means in male and female PEX patients with cataract are not significantly different.

For Glaucoma patients with cataract the levene test’s *p*-value is 0.2747 therefore we have 95% confidence to not reject null hypothesis this concludes that the two sample’s variance are same. Since both assumptions are satisfied, we will perform two sample t-test. From Table 2, below, we can conclude that the two sample means in male and female Glaucoma patients with cataract are not significantly different.

For CHD patients with cataract the levene test’s *p*-value is 0.2444 therefore we have 95% confidence to not reject null hypothesis this concludes that the two sample’s variance are same. Since both assumptions are satisfied, we will perform two sample t-test. From Table 3, below, we can conclude that the two sample means in male and female CHD patients with cataract are not significantly different.

From the three t-test’s result we know that for PEX, Glaucoma and CHD there are no significant different for the mean response in both male and female.

**Table 1:** Two sample t-test for PEX group of both female and male

T test statistic	Degree of freedom	*P*-value	Mean of female	Mean of male

1.3472	594	0.1784	73.33882	72.548

**Table 2:** Two sample t-test for Glaucoma group of both female and male

T test statistic	Degree of freedom	*P*-value	Mean of female	Mean of male

0.6449	170	0.5198	73.442	72.74

**Table 3:** Two sample t-test for CHD group of both female and male

T test statistic	Degree of freedom	*P*-value	Mean of female	Mean of male

1.3725	290	0.171	73.8276	72.625

### Nonparametric analysis

We know that not all of them are follows normal distribution. Therefore we have to perform nonparametric analysis and verify if we get same result. The reason we perform parametric test first is simply because of better power of the test.

From Table 4, above, we can conclude that the nonparametric wilcoxon rank sum test confirm the result from t-test previously. Therefore we can conclude that there are no significant between female and male patients for PEX, Glaucoma and CHD.

**Table 4:** Wilcoxon rank sum test with continuity for PEX, Glaucoma and CHD

PEX		Glaucoma		CHD	
W	*p*-value	W	*p*-value	W	*p*-value

46732.5	0.2625	3883	0.4869	11008.5	0.256

## LOGISTIC REGRESSION ANALYSIS

The response here is binary (0/1) type we have to perform logistic regression analysis in order to modeling the three types of diseases, namely, PEX, Glaucoma and CHD.

The logits of the probabilities of the response are linear function of the attributable variables Xi as in equation (e) for first order covariates only.

(e) $$\mathrm{logit}\left(P\right)=\log\frac{P}{1-P}=\beta_{0}+\beta_{1}x_{1}+...+\beta_{k}x_{k}$$

Here P stands for the probability of the response variable. Betas’ are the coefficients of the attributable variables.

Second order model are shown in equation (f).

(f) $$\log\frac{P}{1-P}=\beta_{0}+\beta_{1}x_{1}+...+\beta_{k}x_{k}+\alpha_{1}x_{1}x_{2}+...+a_{l}x_{k-1}x_{k}$$

In equation (f), alphas’ stand for the coefficients of the 2^nd^ order interaction terms.

By adding higher order interactions we can have higher order logistic regression model. In this paper we will focus on second order model only. Other important references for the readers who will have an interest in logistic regression are in (1, 8-10).

### Female logistic regression analysis

We start with female PEX patients with cataract. The final logistic model is shown in equation (g), below. We considered all attributable variables, namely, age, Glaucoma, CHD and Average IOP (AI) and all possible 2^nd^ order interactions between them. We choose the model by minimum Akaike’s information criterion (AIC) and significance of 2^nd^ order terms.

(g) $$\log\frac{P_{\mathrm{fPEX}}}{{1-P}_{\mathrm{fPEX}}}=-0.8835-0.094\mathrm{age}+10.5\mathrm{Glaucoma}+0.486\mathrm{CHD}-0.104\mathrm{AI}+0.069\mathrm{age}\times \mathrm{AI}-0.453\mathrm{Glaucoma}\times \mathrm{AI}$$

We found that age and AI individual not significant but their interaction effect significantly contribute to the model. The interaction of Glaucoma and AI give negative contribution to the model while individual Glaucoma gives large positive contribution to the model. Glaucoma and CHD individually significantly contribute to the model that means Glaucoma and CHD relates to the risk of PEX. Since both coefficients of Glaucoma and CHD are positive we can conclude that both Glaucoma and CHD will add the risk to have PEX for female patients with cataract.

For female Glaucoma patients with cataract, our final logistic regression model is shown in equation (h), below.

(h) $$\log\frac{P_{\mathrm{fGLAU}}}{{1-P}_{\mathrm{fGLAU}}}=-15.128+7.74\mathrm{PEX}-1.854\mathrm{CHD}+0.634\mathrm{AI}-0.2973\mathrm{PEX}\times \mathrm{AI}$$

Notice that age is not significant individually and with any interaction, therefore we drop this attributable variable. Recall in Figure 3, we mentioned that the fit is not good. Now we know age not affect the risk for female to have Glaucoma when she has cataract. Since we consider age to construct the distribution of Glaucoma, it is not surprise that we get poor distribution fit. PEX gives positive affect to the risk of Glaucoma of female while CHD gives negative affect.

For female CHD patients with cataract, our final logistic regression model is shown in equation (i) below.

(i) $$\log\frac{P_{\mathrm{fCHD}}}{{1-P}_{\mathrm{fCHD}}}=-18.912+0.19\mathrm{age}-0.4626\mathrm{PEX}-2.14\mathrm{Glaucoma}+0.844\mathrm{AI}-0.0092\mathrm{age}\times \mathrm{AI}$$

We found that age, PEX, Glaucoma and AI all individually significant. The interaction of age and AI is also significantly contributed to the model. The interaction of age and AI give negative contribution to the model while individual age and AI both gives positive contribution to the model. Glaucoma individually gives negative contribution to the model which is consistent with we get from equation (h). PEX individually gives positive contribution to the model which is consistent with we get from equation (g).

### Male logistic regression analysis

We start with male PEX patients with cataract. The final logistic model is shown in equation (j) below.

(j) $$\log\frac{P_{\mathrm{mPEX}}}{{1-P}_{\mathrm{mPEX}}}=3.875-0.237\mathrm{age}-15.65\mathrm{Glaucoma}-4.9\mathrm{CHD}-0.298\mathrm{AI}+0.014\mathrm{age}\times \mathrm{AI}-3.74\mathrm{Glaucoma}\times \mathrm{CHD}-0.81\mathrm{Glauco}\times \mathrm{AI}+.35\mathrm{CHD}\times \mathrm{AI}$$

We found that AI individual not significant but the interaction of AI with Glaucoma, AI with age and AI with CHD are all significantly contribute to the model. The interaction of Glaucoma and AI give negative contribution to the model while individual Glaucoma gives large positive contribution to the model. This is same as we found in female model. Glaucoma and CHD individually significantly contribute to the model that means Glaucoma and CHD relates to the risk of PEX. This is also same as in female model. Glaucoma is also positively contributed to the model as in female model. However CHD is negatively contributed to the model. This is opposite with what we found in female model [equation (j)]. The interaction of Glaucoma and CHD is significant to the model which is also different than in model [equation (j)]. We can conclude that Glaucoma will add the risk to have PEX for male patients with cataract but CHD will reduce the risk to have PEX for male patients with cataract.

For male Glaucoma patients with cataract, our final logistic regression model is shown in equation (k), below.

(k) $$\log\frac{P_{\mathrm{mGlau}}}{{1-P}_{\mathrm{mGlau}}}=-12.326+6.24\mathrm{PEX}-11.896\mathrm{CHD}+0.507\mathrm{AI}-5.722\mathrm{PEX}\times \mathrm{CHD}-0.26\mathrm{PEX}\times \mathrm{AI}+0.778\mathrm{CHD}\times \mathrm{AI}$$

Notice that age is not significant individually and with any interaction, therefore we drop this attributable variable. This is same as in female model [equation (k)].

For male CHD patients with cataract, our final logistic regression model is shown in equation (l), below.

(l) $$\log\frac{P_{\mathrm{mCHD}}}{{1-P}_{\mathrm{mCHD}}}=-3.914+0.04\mathrm{age}+0.787\mathrm{PEX}+5.07\mathrm{Glaucoma}-0.06\mathrm{AI}-0.146\mathrm{age}\times \mathrm{Glaucoma}-1.43\mathrm{PEX}\times \mathrm{Glaucoma}+0.3\mathrm{Glaucoma}\times \mathrm{AI}$$

We found that age, PEX, Glaucoma and AI all individually significant. This is same as in female model [equation (l)]. Glaucoma individually gives positive contribution to the model which is opposite with we get from female model [equation (l)]. PEX individually gives positive contribution to the model which is the same as female model [equation (j)].

### Combined logistic regression analysis

From Chapter 2.2 (Probability structure) we know most of the diseases follow Cauchy distribution and from chapter 3.1 (Two sample t-test) and 3.2 (Nonparametric analysis) we know the mean of male and female sample groups are not significantly different. Therefore we decide to combined the two data sets and apply logistic regression analysis.

We start with all PEX patients with cataract. The final logistic model is shown in equation (m) below. Sex is coded as dummy variable 1 for male and 0 for female.

(m) $$\log\frac{P_{\mathrm{aPEX}}}{{1-P}_{\mathrm{aPEX}}}=3.135-0.162\mathrm{age}+14.159\mathrm{Glaucoma}+0.514\mathrm{CHD}-0.317\mathrm{AI}-3.528\mathrm{sex}+0.01\mathrm{age}\times \mathrm{AI}-0.624\mathrm{Glaucoma}\times \mathrm{AI}-2.37\mathrm{Glaucoma}\times \mathrm{sex}+0.23\mathrm{AI}\times \mathrm{sex}$$

We found that AI individual not significant but the interaction of AI with Glaucoma , AI with age and AI with sex are all significantly contribute to the model. The interaction of Glaucoma and AI give negative contribution to the model while individual Glaucoma gives large positive contribution to the model. This is same as we found in the individual model [equation (g)] and 4.6 [equation (m)]. Glaucoma and CHD individually significantly contribute to the model that means Glaucoma and CHD relates to the risk of PEX. This is also same as in individual model. We can conclude that Glaucoma and will add the risk to have PEX for all patients with cataract. Sex is code one represent male therefore male will have less chance to have PEX than female. This is consistent with the observation we made in chapter 2.1 (Descriptive statistic analysis).

For all Glaucoma patients with cataract, our final logistic regression model is shown in equation (n), below.

(n) $$\log\frac{P_{\mathrm{aGlau}}}{{1-P}_{\mathrm{aGlau}}}=-13.97+0.007\mathrm{age}+7.097\mathrm{PEX}-3.38\mathrm{CHD}+0.547\mathrm{AI}+0.677\mathrm{sex}-0.165\mathrm{age}\times \mathrm{CHD}-0.263\mathrm{PEX}\times \mathrm{AI}-1.203\mathrm{PEX}\times \mathrm{sex}+0.576\mathrm{CHD}\times \mathrm{AI}+3.357\mathrm{CHD}\times \mathrm{sex}$$

Notice that age and sex are both not significant individually however they are both significant with interactions. Therefore we drop this attributable variable. This is same as in female model [equation (n)]. The coefficient of sex and age are small means they do not affect the chance to have Glaucoma very much. Notice that age can not be dropped since age and CHD interaction is significant.

For all CHD patients with cataract, our final logistic regression model is shown in equation (o), below.

(o) $$\log\frac{P_{\mathrm{aCHD}}}{{1-P}_{\mathrm{aCHD}}}=-7.59+0.04\mathrm{age}+0.544\mathrm{PEX}+2.24\mathrm{Glaucoma}+0.15\mathrm{AI}+3.4\mathrm{sex}-0.145\mathrm{age}\times \mathrm{Glaucoma}+0.242\mathrm{Glaucoma}\times \mathrm{AI}+3.05\mathrm{sex}\times \mathrm{Glaucoma}-0.183\mathrm{sex}\times \mathrm{AI}$$

We found that age, PEX and AI all individually significant only except for Glaucoma. The coefficient of sex is positive means male have more chance to have CHD than female. This is consistent with what we observed in chapter 2.1 (Descriptive statistic analysis). Both PEX and Glaucoma’s coefficients are positive means to have PEX or Glaucoma will increase the risk to have CHD for the patients with cataract. The coefficient of age is small means age does not affect the opportunity to have CHD a lot.

## CONCLUSIONS

In this paper we start with identify the probabilistic distribution for each disease by comparison of three statistical goodness of fit tests then we will compare the mean response of the age of patients in male and female group through parametric and nonparametric statistical methods.

We analyzed the relationship between PEX, Glaucoma and CHD with all other variables for female, male and both. We utilized the real data of the eye disease over a period of time to perform a statistical analysis for these three diseases and find the relationship and possible interactions between our independent variables. We finally construct logistic regression model for all of the three diseases and by utilizing these three models we can understand the relationship between these diseases and make future forecast for these disease based on the information from the independent attributable variables.

## VOCABULARY

### Cataract

When the normally clear lens inside the eye becomes cloudy or dark and causes blurred vision which is not correctable by ordinary glasses.

### Pseudoexfoliation syndrome

An eye condition that often leads to glaucoma. Called the pseudoexfoliation syndrome because deposits on the surface of the lens look like flakes of dandruff, as if the lens capsule has exfoliated (shed the flakes). It is also called exfoliation syndrome.

### Glaucoma

Permanent loss of vision as a result of damage to the optic nerve in the back of the eye. Treatment for this eye disease is directed toward lowering the eye’s pressure by medications, laser treatment, and/or surgery.

Glaucoma is a group of diseases of the optic nerve involving loss of retinal ganglion cells in a characteristic pattern of optic neuropathy. Raised intraocular pressure is a significant risk factor for developing glaucoma (above 22 mmHg). One person may develop nerve damage at a relatively low pressure, while another person may have high eye pressure for years and yet never develop damage. Untreated glaucoma leads to permanent damage of the optic nerve and resultant visual field loss, which can progress to blindness.

Glaucoma can be divided roughly into two main categories, “open angle” or chronic glaucoma and “closed angle” or acute glaucoma. Angle closure, acute glaucoma appears suddenly and often with painful side effects and so is usually diagnosed quickly, although damage and loss of vision can also occur very suddenly. Open angle, chronic glaucoma tends to progress more slowly and so the patient may not notice it until the disease has progressed quite significantly.

Glaucoma has been nicknamed the “sneak thief of sight” because the loss of visual field often occurs gradually over a long time and may only be recognized when it is already quite advanced. Once lost, this damaged visual field can never be recovered. Worldwide, it is the second leading cause of blindness (1). Glaucoma affects one in two hundred people aged fifty and younger, and one in ten over the age of eighty.

Coronary artery disease (CAD), also called coronary heart disease (CHD), is a condition in which plaque (plak) builds up inside the coronary arteries. These arteries supply the heart muscle with oxygen-rich blood. Plaque is made up of fat, cholesterol (ko-LES-ter-ol), calcium, and other substances found in the blood. When plaque builds up in the arteries, the condition is called atherosclerosis (ATH-er-o-skler-O-sis).
